# Identifying the miRNA Signature Association with Aging-Related Senescence in Glioblastoma

**DOI:** 10.3390/ijms22020517

**Published:** 2021-01-06

**Authors:** Mutharasu Gnanavel, Akshaya Murugesan, Saravanan Konda Mani, Olli Yli-Harja, Meenakshisundaram Kandhavelu

**Affiliations:** 1 BioMediTech Institute, Faculty of Medicine and Health Technology, Tampere University, ArvoYlpönkatu 34, 33520 Tampere, Finland; velan.bio@gmail.com (M.G.); akshaya.murugesan@tuni.fi (A.M.); olli.yli-harja@tuni.fi (O.Y.-H.); 2 Molecular Signalling Lab, Faculty of Medicine and Health Technology, Tampere University, P.O. Box 553, 33101 Tampere, Finland; 3 Department of Biotechnology, Lady Doak College, Thallakulam, Madurai 625002, India; 4 Center for High Performance Computing, Shenzhen Institutes of Advanced Technology, Chinese Academy of Sciences, Shenzhen 518055, China; saravananbioinform@gmail.com; 5 Computational Systems Biology Group, Faculty of Medicine and Health Technology, Tampere University, P.O. Box 553, 33101 Tampere, Finland; 6 Institute for Systems Biology, 1441N 34th Street, Seattle, WA 98109, USA; 7 Science Center, Tampere University Hospital, ArvoYlpönkatu 34, 33520 Tampere, Finland

**Keywords:** glioblastoma, microRNA, senescence, molecular signaling, biochemical pathways

## Abstract

Glioblastoma (GBM) is the most common malignant brain tumor and its malignant phenotypic characteristics are classified as grade IV tumors. Molecular interactions, such as protein–protein, protein–ncRNA, and protein–peptide interactions are crucial to transfer the signaling communications in cellular signaling pathways. Evidences suggest that signaling pathways of stem cells are also activated, which helps the propagation of GBM. Hence, it is important to identify a common signaling pathway that could be visible from multiple GBM gene expression data. microRNA signaling is considered important in GBM signaling, which needs further validation. We performed a high-throughput analysis using micro array expression profiles from 574 samples to explore the role of non-coding RNAs in the disease progression and unique signaling communication in GBM. A series of computational methods involving miRNA expression, gene ontology (GO) based gene enrichment, pathway mapping, and annotation from metabolic pathways databases, and network analysis were used for the analysis. Our study revealed the physiological roles of many known and novel miRNAs in cancer signaling, especially concerning signaling in cancer progression and proliferation. Overall, the results revealed a strong connection with stress induced senescence, significant miRNA targets for cell cycle arrest, and many common signaling pathways to GBM in the network.

## 1. Introduction

Glioblastoma (GBM) is the most common aggressive brain tumor, rendering it incurable by surgery [1,2,3,4]. The mechanisms by which GBM cells migrate and invade the brain is still poorly understood, and leads to limited targeted therapies. Several molecular signaling pathways urge the abnormal growth of cells, such as epidermal growth factor (EGF), platelet derived growth factor (PDGF), vascular endothelial growth factor (VEGF), insulin-like growth factor (IGF), and hepatocyte growth factor/scatter factor (HGF/SF) [5]. It is significant to reveal the interactions of the significant proteins, protein–ncRNA, and protein–peptide to target the crucial signaling pathway in GBM. microRNA signaling communication tends to have an important role in GBM signaling, which needs further validation and understanding [6,7].

On the other hand, small non-coding RNA and microRNA (miRNA) signaling tend to have an essential role in GBM signaling, requiring further validation and understanding [6,7]. miRNAs of about ~22nucleotides in length primarily bind at the 3′ untranslated region of the mRNA, causing degradation or translational repression [8]. GBM patient miRNA microarray data analysis identified 752 miRNAs, in which 115 miRNAs were upregulated and 24 miRNAs were downregulated. Specifically, miR-576-5p, miR-340, and miR-626 were found to have significant overexpression and miR-320, let-7g-5p, miR-7-5P have significant low expression in GBM [9]. The analysis of glioblastoma tissues and glioblastoma cell lines has identified a group of microRNAs that significantly get dysregulated in GBM. It was also noted that miR-221 is strongly upregulated and miR-128, miR-181a, miR-181b, and miR-181c are downregulated in GBM [10]. It has been identified that stable microRNAs circulate in the blood of both healthy individuals and GBM patients. Further, it is identified that the toll-like receptors (TLR) in cancer stem cells interact with mRNA in the central nervous system to influence TLR-4 signaling pathway [11,12].

Senescence is one of the prominent factors in various cancers, including GBM [13,14]. In particular, age-related cancers, such as glioblastoma, have evident connections with cellular aging (otherwise called senescence) [15,16,17]. In The Cancer Genome Atlas (TCGA), a public repository of cancer genomes, GBMs are categorized into three important genetic subtypes: (1) classical, (2) proneural or neural, and (3) mesenchymal, respectively [18]. A careful literature survey suggests that the mesenchymal subgroup has the worst prognosis and recent research focuses on understanding the underlying mechanisms regarding the regulation of mesenchymal GBM. Due to robust gene expression by cancer cells, each subtype greatly varies in terms of its cellular features, genetic contexts, and signaling components [19]. Knowing new signaling components and mechanisms from the senescence pathways will be a crucial step forward in decoding cancer mechanisms. Senescence itself has many complex unknown roots, which are yet to be explored. It was reported that, not a single molecule, but the alterations of a small regulatory module, can induce and maintain a specific phenotypic state in glioma cells [20]. To expand the current view and further understanding on the senescence-associated miRNAs in GBM, we performed a high throughput study using micro array expression profiles from 574 samples. We used a series of computational methods, including statistical analysis of miRNA expression, gene ontology (GO) based gene enrichment, pathway mapping, and annotation from metabolic pathway databases and network analysis. Our study emphasizes the physiological roles of many known and novel miRNAs in cancer signaling, especially GBM, adding finer details regarding the mode of signaling that takes place in cancer progression. Overall observation of our high throughput study mainly emphasizes the involvement of aging related pathways in GBM. Moreover, we focused on senescence as one of the mechanisms responsible for GBM cells, leading to failure on the treatment [21]. Our findings on senescence-related signaling in GBM could lead to decoding the molecular mechanism of GBM, and help discover the possible mode of treatment strategies.

## 2. Results

### 2.1. Significantly Overexpressed miRNAs in GBM

**Figure 1:**
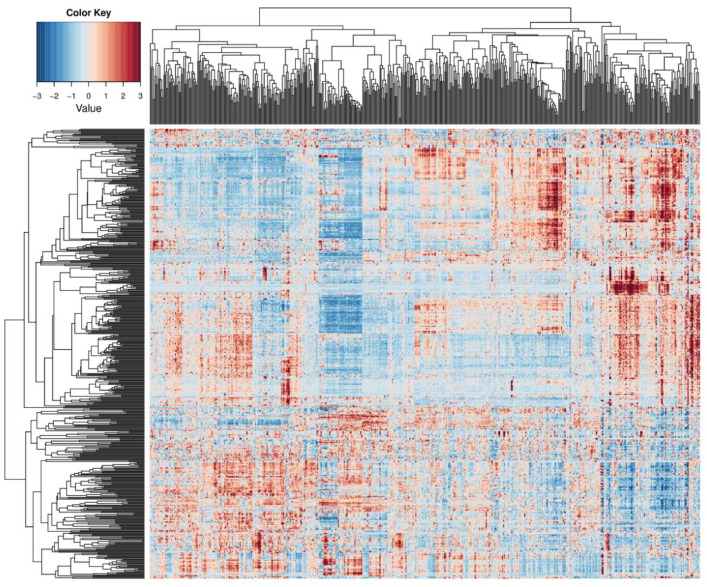
Heat map representation of differentially expressing miRNAs among glioblastoma (GBM) arrays (each array in the map represents an miRNA gene; due to more miRNA genes, gene names are not shown in the figure). Expression levels are scaled into log2 fold change and it scales from −3 (blue) downregulated, to 3 upregulated (brown). Top overexpressing miRNA genes selected from this matrix for further analysis.

We identified novel senescence-related pathways involved in GBM through micro array expression data. This helps to present new signaling interactions governed by miRNA mediated molecular mechanisms. Differentially expressed miRNAs identified were significantly expressed from the matrix (Figure 1), which we further filtered with probe entries, having proper ENTREZ gene mapping and functional annotation. Overexpressed genes were selected based on the log2 fold change with at least two-fold upregulated genes, where about ~534 genes with ≥5 fold upregulated were identified. All ofthese overexpressed genes were used further for the functional annotation search target mapping.

### 2.2. Functional Relevance of Aging in GBM Identified from GO Enrichment Analysis

Reliability and coverage of functional annotation for over expressed genes were further retrieved and confirmed by hypergeometric distribution analysis in two levels: Gene Ontology and Reactome pathway. From the GO based enrichment analysis, we obtained 90 GO terms (Supplementary Table S1) showing potential signaling relevance and molecular signaling in GBM. Out of these, 79 terms are from biological process, nine from molecular function, and two are cellular component related terms. Biological process terms also have significant functionalities related to the development and organization of the central nervous system. In particular, ageneration of neurons (GO: 0048699), central nervous system development (GO: 0007417), and neuron differentiation (GO: 0030182) are a few critical examples. Molecular function-based terms mainly have functional contributions toward signal transduction (GO: 0004871, GO: 0060089) and transcriptional regulation (GO: 0001071, GO: 0003700). Cellular component terms are from cell junction (GO: 0030054) and catalytic complex (GO: 1902494) formation sites.

### 2.3. Reactome Targeted Analysis Captures New Pathways Including Senescence

**Figure 2:**
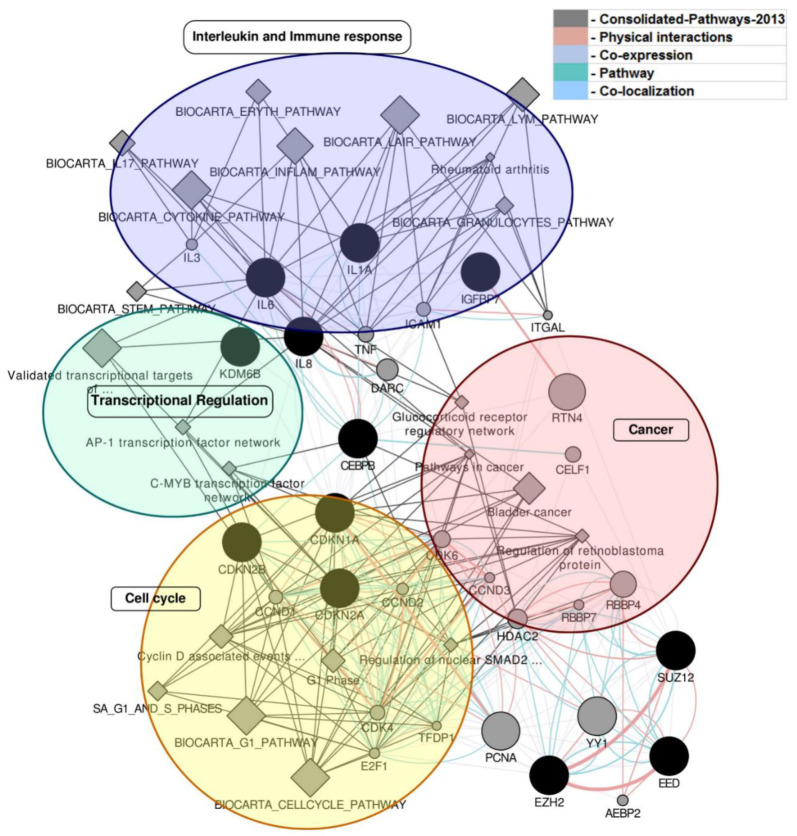
miRNA targeted network of cancer signaling including senescence. Signaling networks of different pathways identified from the analysis of over expressed miRNAs in GBM and their associated targets represented in this network. Black filled circles represent the miRNA input target genes identified from our enrichment analysis and target mapping. Filled Gray circles represent associated genes, having the closest functional relationships with input target genes. Gray diamond shaped nodes represent sub-networks or pathways, having a functional relationship with the genes of interest. Connecting edges are drawn based on the evidence from consolidated pathway databases, physical interactions, co-expression profile, pathways, and co-localization profiles. A network also showing clusters of four different signaling pathways, as highlighted; the top light purple ellipse shows that the most nodes and connections are linked to interleukin and immune response. The small circle on the left, highlighted with light green, shows transcriptional regulation related entries. The light red circle on the right most interestingly shows cancer-linked pathways in genes. The bottom yellow circle shows signaling pathways, which are very essential to cell cycle regulation. Four senescence-linked pathways are present, containing at least 14 genes, as listed in Table 2. Pathway information are selected, based onReactomePathway Database entries, mapping to the identified miRNA target from the network.

Reactome pathway database targeted hypergeometric distribution analysis resulted in13 pathways (Figure 2 and Table 1) having 64 miRNAs, and their associated target genes, including four senescence-related pathways. Identified pathways include many significant connections with tumor phenotypes of the GBM. They are (1) transcriptional activation of cell cycle inhibitor p21 (REACT_346) pathway, where p21 is transcriptionally activated by tumor suppressor protein p53 after DNA damage [22]; (2) DNA damage/telomere stress induced senescence (REACT_169185). This stress induced DNA damage pathway arises from reactive oxygen species (ROS), where concentration increases in senescent cells due to the oncogenic RAS-induced mitochondrial dysfunction [23] or due to the environmental stress, causing double strand breaks (DSBs) in DNA [24]. Additionally, persistent cell division fueled by oncogenic signaling leads to replicative exhaustion, which is manifested in critically short telomeres [25,26].

(3) Formation of senescence-associated heterochromatin foci (SAHF) (REACT_169121) pathway. The processes of these pathways culminate in the formation of senescence-associated heterochromatin foci (SAHF). These foci represent facultative heterochromatin formed in the senescent cells. They contribute to the repression on the proliferation promoting genes and play an important role in the permanent cell cycle exit that finally leads to senescence [27,28]. (4) Binding of TCF/LEF: CTNNB1 to target gene promoters (REACT_264532). Regulation upon the binding of these genesare involved in a diverse range of functions in cellular proliferation, differentiation, embryogenesis, and tissue homeostasis, and include transcription factors, cell cycle regulators, growth factors, proteinases, and inflammatory cytokines [29]. (5) Formation of the beta-catenin: TCF transactivating complex (REACT_264242); this process takes place in chromatin and worksin association with HMG-containing transcription factors that bind to the WNT responsive elements in target gene promoters [30]. (6) SMAD2/SMAD3:SMAD4 heterotrimer regulates transcription (REACT_120734); this complex formation takes place in the nucleus and getsphosphorylated by cyclin dependent kinase CDK8 [31,32]. (7) Transcriptional activation of mitochondrial biogenesis (REACT_264212). Metabolic control of mitochondrial biogenesis happens through the PGC-1 family phosphorylated PPARGC1A (PGC-1alpha) regulatory network [33]. (8) Constitutive signaling by NOTCH1 PEST domain mutants (REACT_160243). NOTCH1 PEST is an intracellular domain, and its mutants are usually behaving like the wildtype NOTCH1, upon ligand binding and proteolytic cleavage mediated activation of signaling. However, after the release of NICD1 fragment of NOTCH1, PEST half-life period, and transcriptional activity, extends through interference with FBXW7 (FBW7)-mediated ubiquitination and degradation of NICD1 [34,35,36]. Identified roles of this pathway include malignant tumor, malignant neoplasm, and primary cancer and T-cell leukemia.

(9) Constitutive signaling by NOTCH1 HD+PEST domain mutants (REACT_160254). Functionality is highly similar to the previous complex and the roles observed were mainly from the T cell acute lymphoblastic leukemia [34]. (10) NOTCH1 intracellular domain regulates transcription (REACT_118780). Activation of NOTCH1 produces NICD1 in response to Delta and Jagged ligands (DLL/JAG) presented in trans, and traffics to the nucleus, where it acts as a transcription regulator in downstream signal transduction [37]. (11) Senescence-associated secretory phenotype (SASP) (REACT_169168). This mediates cellular response to stress arising from RAS and p53 tumor suppressor [38], and DNA damage signaling, and triggers the senescence-associated inflammatory cytokine secretion [39]. (12) POU5F1 (OCT4), SOX2, NANOG activates genes related to proliferation (REACT_264617).POU5F1 (OCT4), SOX2, and NANOG bind elements in the promoters of the target gene playing a role in developmental biology, OCT4 regulatory networks in embryonic stem cells, and embryonal carcinoma cells [40]. (13) Oxidative stress induced senescence (REACT_169436) [23]. “Reactome ID” represents the Reactome curated Pathway Database entry; “Total” is the number of identified miRNA targets from the pathway; “Hypergeometric” is the hypergeometric distribution value for the selected pathway; and the “Description” gives the function of the mentioned pathway.

### 2.4. Semantic Similarity among the Interacting Genes

**Figure 3:**
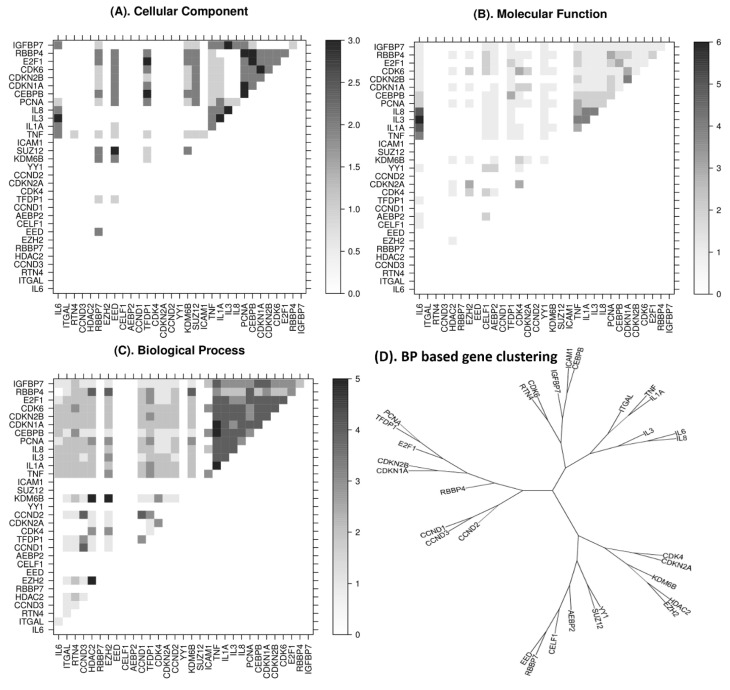
Gene Ontology based semantic similarity among differentially expressed genes. Pairwise comparison of these genes computed using Cytoscape/GeneMANIA plugin (version 3.3.4). Upper diagonal is the semantic similarity matrix excluding self-comparisons. Darker gray-scale represents higher gene ontological relatedness. Pairwise functional similarity graded by the (**A**) cellular component based semantic similarity measured among the genes in pathways observed from the network gene pairs IGFBP7-IL3, RBBP4-PCNA, RBBP4-CEBPB, RBBP4-CDKN1A, CDk6-CDKN1A, E2F1-TFDP1, having some of the strongest connectivity in cellular component based ontology analyses. (**B**) In molecular function (MF) based similarity analysis, ILE family genes are making the strongest pathway connections among themselves and cell-division kinases. (**C**) Compared to cellular component and molecular function analysis, we observed the strongest connectivity in biological process. Immunoglobulins/kinases are one of the strongest subgroups within these gene sets. This indicates the complex and aggressive nature of tumor cells and strong roles of miRNA regulations as well. (**D**) Genes clustered from the biological process-based similarity matrix, which highlights spatial arrangement of the DE genes in relation to the pathways involved.

In total, 31 genes were found as the functional base of annotations resulting from the 90 GO terms identified from the network. The functional relationship among these genes was measured in all three levels of GO annotation: biological process, cellular component, and molecular function. Figure 3 highlights the strong one-to-one connections among these levels. It also indicates the presence of senescence upon the overexpression of miRNAs identified in this study. Further, to examine the interaction of these genes, pathwayanalysis was performed.

### 2.5. Senescence as a Critical Player behind GBM

The analysis revealed that 4 out of 13 miRNA-mediated pathways in GBM were related to senescence (Table 2). We also extracted target gene information for the senescence pathway genes by miRNAs from the above-mentioned Reactome pathways. We identified 13 target genes from senescence regulated by miRNA, which includes **CDKN1A** (Cyclin-Dependent Kinase Inhibitor 1A), **CDKN2A** (cyclin-dependent kinase inhibitor 2A), **CDKN2B** cyclin-dependent kinase inhibitor 2B (p15, inhibits CDK4), **CEBPB** (CCAAT/enhancer binding protein (C/EBP), beta), **CXCL8** (interleukin 8), **EED** (embryonic ectoderm development), **EZH2** (enhancer of zeste homolog 2), **IGFBP7** (insulin-like growth factor binding protein 7), **IL1A** (interleukin 1, alpha), **IL6** (interleukin 6 (interferon, beta 2), **KDM6B** (lysine (K)-specific demethylase 6B), **MIR3074** (microRNA 3074), and **SUZ12** (SUZ12 polycomb repressive complex 2 subunit). Extended network analysis of these target proteins and associated miRNA reveals the signaling pathways from transcriptional regulation, cell cycle control, and cancer related molecular mechanisms (Figure 2 and Figure 3).

## 3. Discussion

Epigenetic variation can alter gene expression or gene regulation, thereby contributing to gliomagenesis. Abnormal metabolism of cancer cells has shown correlation with mutations in genes encoding metabolic enzymes, involved in tricarboxylic acid cycle (TCA). The isocitrate dehydrogenase 1 (IDH1) gene is an example that is known to frequently mutate in different types of cancer and influence EGFR expression [41,42]. Since 2008, sequencing of gliomas has identified IDH1 mutations and the nature of these mutations vary, according to cancer types. Interestingly, in some types of cancer, these mutations are rare and not observed. The metabolic enzymes encoded by IDH1 genes convert isocitrate to α-ketoglutarate, producing NADPH, and participate in glucose sensing, lipid metabolism, and oxidative respiration [43], and the activity of these enzymes are shown to protect against replicative senescence by reducing oxidative DNA damage. In the post-genomic era, the abnormal gene expressions linked with various tumors, including glioma, were studied by miRNA sequence analysis. The miRNAs are small endogenous non-coding RNAs, composed of 18–23 nucleotides that regulate gene expression in the cells. The results of RNA sequence analysis indicate that the subset of miRNAs is deregulated in glioma that plays a vital role in proliferation, invasion, and migration [44]. The differential expression analysis of 574 samples from the TCGA database was performed. The differentially expressed miRNAs from glioma samples were considered and subjected to further validation by hypergeometric distribution analysis. The hypergeometric distribution analysis uses gene ontology enrichment and Reactome pathways direction to identify potential signaling relevance and molecular signaling in GBM. Biological processes related GO terms include many interesting terms having high correlation with cellcycle and aging functional pathways. Aging related terms includes regulation of programmed cell death (GO: 0043067), apoptotic process (GO: 0042981), cell death (GO: 0010941), cell cycle (GO: 0051726), cell differentiation (GO: 0045595). List of Reactome database pathway entries identified from the mapping and annotation of over expressed miRNAs in GBM micro array data. There are four pathways observed from senescence related mechanisms, which supports the existing hypothesis about the link of GBM with aging. We used those senescence-linked pathways for further detailed study using network analysis. Through our observations, 31 genes were found as the functional base of annotations resulting from the 90 GO terms identified from the network based on the GO annotation, which includes biological process, cellular component, and molecular function. The results from the computational investigation of tumor and non-tumor samples indicated the presence of senescence upon the overexpression of miRNAs identified, further biochemical and mutational experiments will shed light on the influence of certain genes in modulating molecular mechanism of senescence related signaling pathways.

## 4. Materials and Methods

### 4.1. GBM Data Retrieval

Agilent single channel (green) microarray data of 574 samples each containing 12,033 probes was retrieved from The Cancer Genome Atlas (TCGA) repository [18] using “Data matrix and Bulk download” facility. We used Level 1 data (having raw signals) and Level 2 data containing normalized signals of miRNA expression, per probe, set for each participant’s tumor sample.

### 4.2. Microarray Data Processing and miRNA Differential Expression Analysis

**Figure 4:**
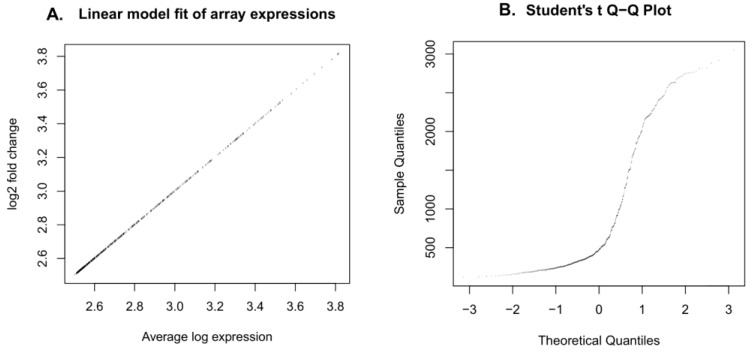
Linear fitting and statistical testing of normalized miRNA expression data. Linearmodel fitting performed for all ofthe GBM miRNA arrays with the same probes used in this analysis. (**A**) Correlation between observed log2 fold expressions plotted against average expression in y and x axes, respectively. (**B**) Students Quantile–Quantile plot showing the quantiles of data sample against the theoretical quantiles of student’s t distribution calculated for the sample.

Primary array data from 574 samples were merged into an expression matrix and pre-processing was performed by normalization and differential expression analysis using series of R/Bioconductor [45] packages. Agilent Microarray probes were mapped into gene names using the AgiMicroRna [46] package. Data analysis, linear model for microarray, and RNA-seq data (limma) [47] from Bioconductor was used to read all ofthe expression levels into a data matrix. Quantile normalization was used between array normalization methods, while background offset was set to 50. Normalized matrix was converted into log2 fold to maintain uniform scale across arrays and genes. Linear model fit was performed for the log2 scaled matrix followed by the Empirical Bayes fitting to maintain the stability of the results (Figure 4A,B). Agilent miRNA annotation for all ofthe miRNAs and associated targets were collected from miRbase [48] and TargetScan [49] using the RmiR Bioconductor package. Top upregulated genes were selected based on the adjusted *p*-value cut-off of 0.05 from the linear model fit matrix. Adjustment method “BH” was used to control the expected false discovery rate (FDR) with the specified *p*-value, which is the most appropriate criteria for microarray studies.

### 4.3. GO Annotation-Based Enrichment

Gene Ontology [50] annotation based analysis was carried out for upregulated miRNAs from the normalized matrix using GOStats package [51]. GOStats package has set of tools to use with GO and microarray data, and it is used for a variety of basic data manipulation, hypothesis testing, and calculations. All three annotation levels for biological process, molecular function, and cellular component were implied to get the relationships. p-value threshold of 0.05 was used without any adjustments, and the minimum number of genes required from any identified pathway was set to 5 to collect the GO terms. We performed the hypergeometric test for all ofthe genes having annotations and associated GO terms. Microarray probes that had no ENTREZ gene ID or GO term association were filtered out. The hypergeometric test, also called hypergeometric distribution, is a probability distribution describing the number of successes, selected from a population with no replacement. Based on this distribution, we selected significant GO terms associated with overexpressed miRNAs.

### 4.4. Pathway Mapping Using Reactome

We performed hypergeometric distribution analysis targeting the Reactome [52] pathway with a similar approach applied to the GO term annotation search. Instead of searching for GO terms for overexpressed miRNAs, we searched for appropriate pathway associations for the selected genes. Statistical test for the association of selected overexpressed miRNA with ENSEMBL genes was identified using the CORNA package [53]. Significantly overexpressed genes were used as sample, and associations between these genes and miRNAs were obtained from miRbase [48]. For the mapping of miRNA transcripts to ENSEMBL genes, we used the mapping function from the CORNA package. For every miRNA having association with at least one target gene from any pathway, this function counts the total number of genes and associates with it. A hypergeometric test was applied to infer whether this miRNA is more likely to associate significantly with genes from Reactome pathways. The minimum population threshold of one gene was set for the search and result, based on the hypergeometric value. Reactome pathways having a hypergeometric distribution value of ≤0.4 were selected, and the results were saved as separate files, having information about the miRNA target genes, Reactome pathways, transcript to gene mapping, and miRNA to gene mapping.

### 4.5. Hypergeometric Test

Minimum population threshold for a gene was set to five, and the result was sorted based on the hypergeometric value. A set of significant genes were defined based on the p-values less than the threshold. The hypergeometric test evaluated the pathway of interest that contained more significant genes, compared to those outside the pathway than expected. For a pathway with *x* significant genes, the p-value of enrichment of the pathway *p* with *m* genes is calculated by the following Equation (1)

(1) $$PHT=\sum_{j=x}^{K_{s}}\frac{\left(\begin{array}{c} K-m\\ K_{s}-j \end{array}\right)\left(\begin{array}{c} m\\ j \end{array}\right)}{\left(\begin{array}{c} K\\ K_{s} \end{array}\right)}$$

where *K_s_* is the length of the significant genes list, *K* is total number of the genes for the evaluation. This test assumes that the list of significant genes is random and conditions on a fixed pathway. This is a one-sided test thatallows to check if the pathway is enriched/over-represented within the list of most significantly associated genes for the given phenotype [54]. Reactome pathways having hypergeometric distribution (*PHT*) values of ≤0.05 were selected. Results include information about the miRNA target genes, Reactome pathways, transcript to gene mapping, and miRNA to gene mapping.

### 4.6. Network Analysis of miRNA Targets

All ofthe genes of Reactome pathways identified from the hypergeometric test were used along with their targeting miRNAs for network analysis. Network analysis was carried out in the Cytoscape [55] environment, using the geneMANIA plugin [56]. Attributes included for network construction were co-expression, co-localization, genetic interactions, pathways, physical interactions, predicted, and shared protein domain-based interactions. All ofthe evidence for these attributes were collected from consolidated pathways, drug-interactions, InterPro database, miRNA-targets predictions, and transcriptional factor targets. For every input gene, the top 20 most connected genes and top 20 attributes were searched.The resulted network had 48.39% of attributes from consolidated pathways, 25.06% from physical interactions, 14.07% based on co-expression, 10.31% from common pathways, and 2.17% based on co-localization evidence. 

### 4.7. Quantitative Measurement of Functional Relativeness

To express the quantitative functional relationships among the GO terms observed (Supplementary Table S1), in relation to the gene entities in the signaling network, a semantic similarity analysis was performed using Python module, FastSemSim [57]. In total, 32 genes connected to 90 GO terms from the network were used for this analysis. Resnik similarity measure and Best Match Average (BMA) mixing strategy implemented in FastSemSim were used. Resnik similarity [58] calculated the similarity of terms (*t*1 and *t*2), based on the information content (IC) of the maximum value of informative common ancestor (MICA) [57,58,59].

sim Res (*t*1, *t*2) = IC [MICA (*t*1, *t*2)] (2)

where, MICA (*t*1, *t*2) = arg max, I (*tj*) and *tj*Aancestors (*t*1, *t*2) and IC(*t*) = −log[*p*(*t*)]. BMA provides the average similarity between the best-matching terms [60]. Resnik measure and BMA mixing strategy is a preferred combination and are often identified as the best measures [61,62]. Semantic similarity values were computed for all pairs of genes and the similarity matrix was used to construct the interaction tree. Its representation was plotted using iTOL [63].

## 5. Conclusions

RNAseq analysis of cancer data can aid in identifying tumor-related miRNA that function as an oncogene or tumor suppressor. In our analysis, the levels of certain miRNA in glioma tissues were notably underexpressed or overexpressed compared to corresponding non-tumor tissues. The differentially expressed miRNA targeted genes can be studied by using cellular assays to understand the blocking of cell cycle and proliferation. Altogether, these experiments reveal that certain miRNA loss facilitates malignant phenotype of glioma cells through a specific biochemical pathway. GBM have strong communications with aging induced stress and it is common among elderly males, which get aggressive as age increases. In the present work, we performed a differential expression analysis between all 574 glioblastoma tumor samples obtained from the TCGA database, with the goal of identifying significant associations between miRNA targets, their enriched functional categories, and tumor etiologies. Our analysis revealed the overexpressed Differentially Expressed Genes (DEGs) and GO annotations related to many senescence-linked molecular mechanisms in GBM. A major proportion of the genes identified as miRNA targets are the inhibitors of cell cycle and proliferation. Blocking such inhibitors will make cells into an aggressive proliferate condition, and virtually immortal, which is the hallmark mechanism of any tumor, including GBM. Observation of other cancer pathways, including bladder cancer and retinoblastoma in the network suggests the possible overlapping of signals among these cancer types.

## Figures and Tables

**Table 1:** Reactome pathways identified for overexpressed miRNA using hypergeometric testing.

Reactome ID	Total	Hypergeometric	Description
REACT_346	1	0.0746269	Transcriptional activation of cell cycle inhibitor p21
REACT_169185	1	0.0746269	DNA damage/telomere stress induced senescence
REACT_169121	1	0.0746269	Formation of senescence-associated heterochromatin foci (SAHF)
REACT_264532	2	0.1442038	Binding of TCF/LEF:CTNNB1 to target gene promoters
REACT_264242	2	0.1442038	Formation of the beta-catenin:TCF transactivating complex
REACT_120734	5	0.3256145	SMAD2/SMAD3:SMAD4 heterotrimerregulatestranscription
REACT_264212	16	0.3404253	Transcriptional activation of mitochondrial biogenesis
REACT_160243	6	0.3778924	Constitutive signaling by NOTCH1 PEST domain mutants
REACT_160254	6	0.3778924	Constitutive signaling by NOTCH1 HD+PEST domain mutants
REACT_118780	6	0.3778924	NOTCH1 intracellular domain regulates transcription
REACT_169168	6	0.3778924	Senescence Associated Secretory Phenotype (SASP)
REACT_264617	6	0.3778924	POU5F1 (OCT4) SOX2 NANOG activate genes related to proliferation
REACT_169436	6	0.3778924	Oxidative-stress-induced-senescence

**Table 2:** Senescence linked pathways and miRNA targeting genes in GBM.

Reactome ID	Pathway Description	Genes
REACT_169185	DNA Damage/Telomere Stress Induced Senescence	ENSG00000124762 (cyclin-dependent kinase inhibitor 1A: CDKN1A)
REACT_169121	Formation of Senescence-Associated Heterochromatin Foci (SAHF)	ENSG00000124762 (cyclin-dependent kinase inhibitor 1A: CDKN1A)
REACT_169168	Senescence-Associated Secretory Phenotype (SASP)	ENSG00000115008 (interleukin 1: IL1A)
		ENSG00000136244 (interleukin 6: IL6)
		ENSG00000147883 (cyclin-dependent kinase inhibitor 2B: CDKN2B)
		ENSG00000163453 (insulin-like growth factor binding protein 7: IGFBP7)
		ENSG00000169429 (chemokine (C-X-C motif) ligand 8: CXCL8)
		ENSG00000172216 (CCAAT/enhancer binding protein: CEBPB)
REACT_169436	Oxidative Stress Induced Senescence	ENSG00000074266 (embryonic ectoderm development: EED)
		ENSG00000106462 (enhancer of zeste 2 polycomb repressive complex 2 subunit: EZH2)
		ENSG00000132510 (lysine (K)-specific demethylase 6B: KDM6B)
		ENSG00000147889 (cyclin-dependent kinase inhibitor 2A: CDKN2A)
		ENSG00000178691 (SUZ12 polycomb repressive complex 2 subunit: SUZ12)
		ENSG00000207617 (microRNA 3074: MIR3074)

## Data Availability

The data used in this paper is available from public repositories.

## Supplementary Materials

Supplementary materials can be found at https://www.mdpi.com/1422-0067/22/2/517/s1.
