# Supplementary material for: Identifying the miRNA Signature Association with Aging-Related Senescence in Glioblastoma
Source: Int J Mol Sci. 2021 Jan 6;22(2):517. doi: 10.3390/ijms22020517 (PMC7825621; doi:10.3390/ijms22020517)
Supplement: Supplementary file 1 [file ijms-22-00517-s001.pdf]

Supplementary Table 1. Gene Ontology (GO) annotations of miRNA targeting cellular signaling retrieved using hyper geometric testing. Identified GO terms, p-value level of ontology annotation (biological process, molecular process and cellular component) along with functional description are given from the network analysis. Lower the P-value gives better reliability of functional relevance for the identified in the network.

GO Term	P-value	Description	Ontology
GO:0050794	0.0000	regulation of cellular process	biological process
GO:0050789	0.0000	regulation of biological process	biological process
GO:0065007	0.0000	biological regulation	biological process
GO:0043067	0.0002	regulation of programmed cell death	biological process
GO:0042981	0.0002	regulation of apoptotic process	biological process
GO:0010941	0.0002	regulation of cell death	biological process
GO:0051254	0.0005	positive regulation of RNA metabolic process	biological process
GO:0006468	0.0006	protein phosphorylation	biological process
GO:1902680	0.0008	positive regulation of RNA biosynthetic process	biological process
GO:0010562	0.0010	positive regulation of phosphorus metabolic process	biological process
GO:0045937	0.0010	positive regulation of phosphate metabolic process	biological process
GO:0031399	0.0010	regulation of protein modification process	biological process
GO:0051726	0.0011	regulation of cell cycle	biological process
GO:0045595	0.0011	regulation of cell differentiation	biological process
GO:0042327	0.0011	positive regulation of phosphorylation	biological process
GO:0045893	0.0011	positive regulation of transcription, DNA-templated	biological process
GO:0031401	0.0013	positive regulation of protein modification process	biological process
GO:0010628	0.0013	positive regulation of gene expression	biological process
GO:0051247	0.0016	positive regulation of protein metabolic process	biological process
GO:0044699	0.0019	single-organism process	biological process
GO:0032270	0.0020	positive regulation of cellular protein metabolic process	biological process
GO:0033043	0.0022	regulation of organelle organization	biological process
GO:0045944	0.0025	positive regulation of transcription from RNA polymerase II promoter	biological process
GO:0044763	0.0026	single-organism cellular process	biological process
GO:1901701	0.0032	cellular response to oxygen-containing compound	biological process
GO:0080134	0.0032	regulation of response to stress	biological process
GO:0010648	0.0042	negative regulation of cell communication	biological process
GO:0023057	0.0043	negative regulation of signaling	biological process
GO:0007268	0.0046	synaptic transmission	biological process
GO:0007167	0.0049	enzyme linked receptor protein signaling pathway	biological process
GO:0042325	0.0052	regulation of phosphorylation	biological process
GO:0018193	0.0053	peptidyl-amino acid modification	biological process
GO:0051130	0.0058	positive regulation of cellular component organization	biological process
GO:0071495	0.0063	cellular response to endogenous stimulus	biological process
GO:0060284	0.0064	regulation of cell development	biological process
GO:0043085	0.0073	positive regulation of catalytic activity	biological process
GO:0009968	0.0077	negative regulation of signal transduction	biological process
GO:0016192	0.0079	vesicle-mediated transport	biological process
GO:2000026	0.0086	regulation of multicellular organismal development	biological process
GO:0048699	0.0089	generation of neurons	biological process
GO:1901068	0.0097	guanosine-containing compound metabolic process	biological process
GO:0046039	0.0100	GTP metabolic process	biological process
GO:0001932	0.0106	regulation of protein phosphorylation	biological process
GO:1901069	0.0108	guanosine-containing compound catabolic process	biological process
GO:0006184	0.0110	GTP catabolic process	biological process

GO:0007417	0.0111	central nervous system development	biological process
GO:0051172	0.0119	negative regulation of nitrogen compound metabolic process	biological process
GO:0051094	0.0123	positive regulation of developmental process	biological process
GO:0030182	0.0126	neuron differentiation	biological process
GO:0048585	0.0145	negative regulation of response to stimulus	biological process
GO:0045892	0.0148	negative regulation of transcription, DNA-templated	biological process
GO:0045934	0.0150	negative regulation of nucleobase-containing compound metabolic process	biological process
GO:0051336	0.0178	regulation of hydrolase activity	biological process
GO:0009790	0.0178	embryo development	biological process
GO:0007169	0.0185	transmembrane receptor protein tyrosine kinase signaling pathway	biological process
GO:0070647	0.0185	protein modification by small protein conjugation or removal	biological process
GO:0051253	0.0186	negative regulation of RNA metabolic process	biological process
GO:0009890	0.0193	negative regulation of biosynthetic process	biological process
GO:0031327	0.0219	negative regulation of cellular biosynthetic process	biological process
GO:0051345	0.0221	positive regulation of hydrolase activity	biological process
GO:1902679	0.0244	negative regulation of RNA biosynthetic process	biological process
GO:0007010	0.0267	cytoskeleton organization	biological process
GO:0010629	0.0275	negative regulation of gene expression	biological process
GO:2000113	0.0291	negative regulation of cellular macromolecule biosynthetic process	biological process
GO:0070848	0.0295	response to growth factor	biological process
GO:0022603	0.0305	regulation of anatomical structure morphogenesis	biological process
GO:0031329	0.0321	regulation of cellular catabolic process	biological process
GO:1901700	0.0335	response to oxygen-containing compound	biological process
GO:0071363	0.0347	cellular response to growth factor stimulus	biological process
GO:0009719	0.0350	response to endogenous stimulus	biological process
GO:0010558	0.0353	negative regulation of macromolecule biosynthetic process	biological process
GO:0061024	0.0367	membrane organization	biological process
GO:0007267	0.0412	cell-cell signaling	biological process
GO:0045184	0.0421	establishment of protein localization	biological process
GO:0009894	0.0421	regulation of catabolic process	biological process
GO:0048666	0.0425	neuron development	biological process
GO:0009725	0.0442	response to hormone	biological process
GO:0051338	0.0452	regulation of transferase activity	biological process
GO:0031175	0.0483	neuron projection development	biological process
GO:0005515	0.0000	protein binding	molecular function
GO:0043565	0.0010	sequence-specific DNA binding	molecular function
GO:0008270	0.0011	zinc ion binding	molecular function
GO:0001071	0.0063	nucleic acid binding transcription factor activity	molecular function
GO:0003700	0.0064	sequence-specific DNA binding transcription factor activity	molecular function
GO:0005488	0.0127	binding	molecular function
GO:0016301	0.0317	kinase activity	molecular function
GO:0004871	0.0397	signal transducer activity	molecular function
GO:0060089	0.0397	molecular transducer activity	molecular function
GO:0030054	0.0052	cell junction	cellular component
GO:1902494	0.0466	catalytic complex	cellular component
